# Effects of phloretin on oxidative and inflammatory reaction in rat model of cecal ligation and puncture induced sepsis

**DOI:** 10.1186/s40199-016-0154-9

**Published:** 2016-05-06

**Authors:** Mehdi Aliomrani, Mohammad Reza Sepand, Hamid Reza Mirzaei, Ali Reza kazemi, Saeid Nekonam, Omid Sabzevari

**Affiliations:** Toxicology and Poisoning Research Centre, Tehran University of Medical Sciences, Tehran, Iran; Department of Pharmacology and Toxicology, Faculty of Pharmacy, Tehran University of Medical Sciences, P. O. Box, 1417614411, Tehran, Iran; Department of Immunology, Faculty of Medicine, Tehran University of Medical Sciences, Tehran, Iran; Department of Anatomy, Faculty of Medicine, Tehran University of Medical Sciences, Tehran, Iran; Drug design and discovery Research Centre, Tehran University of Medical Sciences, Tehran, Iran

**Keywords:** Phloretin, Sepsis, NF-ĸB, TNF- α, Oxidative stress, Antioxidants

## Abstract

**Background:**

Sepsis is a debilitating systemic disease and described as a severe and irregular systemic inflammatory reaction syndrome (SIRS) against infection. We employed CLP (Cecal Ligation and Puncture) model in rats to investigate anti-inflammatory and antioxidant effects of phloretin, as a natural antioxidant agent, and its protective effect on liver tissue damage caused by sepsis.

**Methods:**

Male Wistar albino rats were randomly divided into three groups: sham group, CLP induced sepsis group and phloretin treated CLP group. Sepsis was induced by CLP method. 50 mmol/kg Phloretin was administered intraperitoneally in two equal doses immediately after surgery.

**Results:**

It was observed that blood urea nitrogen (BUN) and tumor necrosis factor alpha (TNF-α) levels were dramatically increased in the CLP induced sepsis group (43.88 ± 1.905 mg/dl, 37.63 ± 1.92, respectively) when compared to the sham group. Moreover, tissue Glutathione (GSH) and liver nuclear factor ĸB (NF-ĸB p65) transcription factor values were higher in CLP induced sepsis group. This elevation was considerably reduced in the phloretin treated CLP group. No significant differences were observed in serum creatinine and creatinine phosphokinase levels.

**Conclusions:**

The present study suggested that phloretin, as a natural protective agent, act against tissue damages introduced following the experimental sepsis induced model, likely caused by free oxygen radicals.

## Background

Sepsis is a common and expensive condition especially in the elderly, which is associated with a very high mortality rate (40 to 60 %) [1, 2]. In spite of advances in critical care treatment of the disease such as invasive surgical therapy and chemotherapy, and better understanding of its pathophysiology, the fatal frequency has not changed significantly over the past 35 years, even in developed countries. Sepsis is described as a severe and irregular systemic inflammatory reaction syndrome (SIRS) against infection [3]. Bacterial lipopolysaccharide endotoxins trigger macrophages and enhance the release of pro-inflammatory cytokines, hence play a pivotal role in the development of systemic inflammatory response. Free oxygen radicals are among important mediators responsible for the inflammatory response. They disrupt cell membranes by rising lipid peroxidation, inhibit ATP synthesis in the mitochondria, and may lead to oxidative damage to DNA and proteins [4].

The systemic inflammatory stress response such as sepsis, septic shock, and multiple organ dysfunction syndromes are conditions of severity and deterioration of essential organ function [5, 6]. It has been documented that a relation exists between oxidative stress and peritoneal sepsis [7]. Antioxidant agents might counter the toxicity of reactive oxygen species and reactive nitrogen species [8, 9], thus free radical ablation might be complementary in the clinical management of sepsis-induced multiple organ failures [10].

Secondary plant metabolites are a vast group of chemicals that are synthesized from carbohydrates, fatty acids and amino acids [11]. Flavonoids are polyphenolic compounds in a natural manner and represent one of the most extensive ingredients in fruits, vegetables, nuts, tea and coffee [11], as well as in herbal medicine [12]. Flavonoids are made up of flavones, flavonols, flavanones, chalcones, anthocyanins, and isoflavones. Flavonoids are well known for their role as an anti-inflammatory and antioxidant agents and shown to have health encouraging, chemopreventive, and disease-preventing characteristics [13, 14].

Chalcones contain 2 benzene rings which are bound together by an unsaturated carboxyl group. They are the secondary metabolite of plants with a chemical structure similar to curcumin [15]. By now, chalcones proven to have many therapeutic characteristics such as antifungal, anti-leishmania, anti-bacterial, anti-malarial and chemopreventive activities, in which part of these properties disclosed to antioxidant activity and chelating the metallic ions [16, 17]. Phloretin is a dihydrogen chalcone flavonoid which primarily extracted from apple [18]. Many studies have demonstrated the pharmacophore responsible region for the anti oxidative activity of phloretin [19, 20]. However, no detailed investigation has described the role of phloretin in sepsis induced by CLP model. This study was designed to investigate protective effect of phloretin against sepsis-induced oxidative organ damage using biochemical parameters, such as nuclear factor kappa-light-chain-enhancer of activated B cells (NF-ĸB), tumor necrosis factor–alpha (TNF-*α*) and tissue glutathione (GSH) levels as well as the histopathological examination of liver tissues.

## Methods

### Chemicals

2′, 4′, 6′, 4 Tetrahrdroxydihydrochalcone (phloretin) was obtained from Sigma-Aldrich Company (St. Louis, MO, USA). All solvents including ethanol, tween 80 and DMSO were supplied by Merck Company (Darmstadt, Germany). TNF-α assay kit were purchased from Biosource Europe, S.A. (Nivelles, Belgium). P65 NF-ĸB transcription factor kit was obtained from Cayman chemical (USA).

### Animals

Animal experiments were carried on in the light of the protocol of Ministry of Health & Medical Education Convention for the Protection of vertebrate animals used for experimental and other scientific goals, and the protocol was accepted by the Ethics Committee of the Tehran University of Medicine Sciences (TUMS), Tehran, Iran. Male Wistar albino rats, weighing 200-250 g, were included in the study. The rats were kept in a temperature controlled (22 ± 1 °C) room with a 12:12 h alternating light/dark cycle in Animal Care Center, Faculty of Pharmacy, TUMS.

### Cecal Ligation and Puncture (CLP) as a sepsis model

General anesthesia was applied to the rats using ketamine HCl (100 mg/kg) and xylazine HCl (10 mg/kg) via intraperitoneal route. The subjects were immobilized on the operation table in the supine position and the abdominal skin was shaved totally, and laparotomy was carried out through a 2 cm midline incision. Poly microbial sepsis was induced by the CLP technique described previously [21]. The cecum was isolated through laparotomy, and the ascending colon was gently touched downward to fill the cecum with feces. The cecum was ligated below the ileocecal valve using 3.0 silk thread, and the ventral side was perforated twice using No.21 gauge needle to let fecal contents spread into the peritoneum. Then, the abdomen was closed with two layers continuous suture using 3.0 silk thread. Rats were resuscitated with normal saline (1 ml/100 g body weight given subcutaneously) at the end of the operation. In the sham group, the cecum was explored but CLP was not applied. The rats were monitored in room temperature of 22 °C, with controlled humidity and light and allowed standard food and drinking water till 12 h after the operation. All the rats were sacrificed 24 h after the operation.

## Experimental protocol

The rats were randomly subdivided into 4 groups of 7 rats. Group I (Sham group): operative procedure was applied, but cecal ligation perforation was not performed. Group II (CLP group): sepsis was induced using the cecal ligation and perforation (CLP) method. Group III (Phloretin group): sepsis was induced using the CLP method and 50 mmol/kg phloretin was administered via the intraperitoneal route in two equal doses immediately after surgery and at the post-operative 12th hour and finally, control group which peritoneally received dimethyl sulfoxide as phloretin solvent [15, 22].

### Blood collection and determination of biochemical parameters

Blood samples collected through a cardiac puncture, centrifuged at 1000 g for 5 min and serum was separated. The samples were then transferred into Eppendorf tubes and stored at 4 °C within 1 h after collection for biochemical assay according to the instructions provided by the manufacturers.

Serum creatinine concentration was measured using Jaffe method [21]. The rest of serum was stored at -70 °C for further analysis. BUN and creatine phosphokinase (CPK) were determined by UV method using auto analyzer (COBAS Integra C111; Roche Diagnostics, Switzerland).

### Measurement of serum TNF-α level

Serum level of TNF-α, as an inflammatory cytokine, was assayed according to the manufacturer’s instruction using an enzyme-linked immunosorbent assay (ELISA) kit from BioSource Europe. The concentration of TNF-α was calculated from the standard curve and results were presented as pg/ml.

### Immunoassay of NF-ĸB P65

To investigate liver NF-ĸB activity, p65 Transcription Factor Assay kit (Cayman chemical) was employed. NF-ĸB activity was measured calorimetrically according to the manufacturer’s instructions. For preparation of hepatic cells nuclear fraction, frozen liver tissue was homogenized in lysis buffer with 2 % Triton X-100 and aprotinin, and centrifuged at 1000 g for 5 min. The pellet was then resuspended in 5 ml ice-cold 1X Nuclear extraction PBS/Phosphatase inhibitor solution and centrifuged at 300xg for 5 min at 4 °C, and the aliquot of the nuclear extract was used for the measurement. Total proteins were quantified by Lowry’s method (Lowry et al. 1951). Briefly, 10 μl of samples were added to the appropriate well that contained its relevant complete transcription factor buffer (CTFB). The plate was incubated overnight at 4 °C, and the wells were washed with 1x washing buffer five times. Primary antibody was then added to the appropriate well, and the plate was incubated for 1 h at 37 °C. Subsequently, the horseradish peroxidase (HRP) linked secondary antibody was added and the plate was incubated for 30 min at 37 °C. Following addition of the substrate, plates were incubated for 45 min at room temperature, the reaction was stopped and samples were measured spectrophotometerically at 450 nm. Each phosphoprotein absorbance was corrected by the negative control and was normalized by its relevant NF-ĸB p65 absorbance.

### Tissue GSH level

The level of GSH was determined by the method explained previously by Sedlak and Lindsay [22]. This assay is based on the production of yellow color when 5, 5-dithionitrobisbenzoic acid (DTNB) is added to compounds containing sulphydryl groups. The liver tissue was homogenized in 0.02 M EDTA and mixed for 10–15 min with an equal volume of 10 % TCA. It was then centrifuged at 10,000 g for 10 min. 1 ml of the supernatant was mixed with 0.5 ml of Ellman’s reagents (19.8 mg of DTNB in 100 ml of 0.1 % sodium nitrate) and 3 ml of phosphate buffer (0.2 M, pH 8.0). The absorbance was immediately recorded at 412 nm on a spectrophotometer. Results were expressed as μmol GSH/g tissue [23].

## Histopathological examination

Liver tissues were fixed in a 10 % formalin solution. They were washed with PBS buffer overnight and were slowly dehydrated by crossing through the solutions by enhancing the concentrations of alcohol. Subsequently, the tissue samples were placed in paraffin and were fixed in blocks. Then sections with 5-6 μm thickness were obtained from the tissue samples placed on slides. Finally, slides were stained by hematoxylin-eosin and examined under a light microscope (Olympus BHX51; Tokyo, Japan) at X40 magnification.

## Statistical analysis

Statistical analysis was performed using a GraphPad Prism 3.0 (GraphPad Software, San Diego; CA; USA). Data were evaluated by one-way analysis of variance (ANOVA) and Tukey’s test was used for pairwise comparisons. All data were expressed as means ± SEM. Values of p ≤ 0.05 were considered as statistically significant.

## Results

### Blood Urea Nitrogen (BUN)

**Fig. 1:**
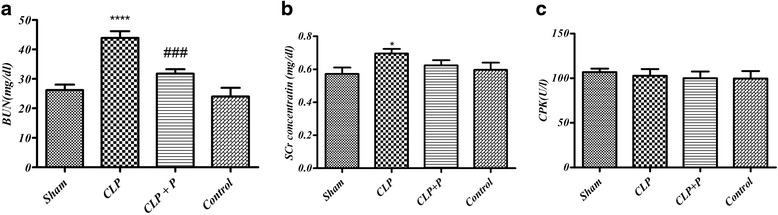
**a** Serum level of BUN in Sham (negative control), CLP (cecal ligation and puncture as a positive control) and CLP + P (CLP rats treated with Phloretin) in 24 h after sepsis induction by CLP. Data are presented as mean ± SEM. CLP (cecal ligation and puncture) vs. Sham group (****) *p* < 0.0001, CLP+ phloretin vs. CLP group (##) *p* < 0.01. **b** and **c**. Serum level of Creatinine and creatinine phosphokinase, respectively (*n* = 7 per group). Data are presented as mean ± SEM. CLP (cecal ligation and puncture) vs. Sham group (*) *p* < 0.05

Our findings showed that cecal ligation and puncture caused a significant increase in serum BUN level (43.88 ± 1.905 mg/dl) compared with the sham group (26.17 ± 2.303), *p* < 0.01. Following treatment with phloretin (CLP + P), serum BUN levels were decreased (31.77 ± 1.514 mg/dl), in comparison with CLP group (*p* < 0.01), Fig. 1a.

### Serum creatinine level (Scr)

Serum creatinine mildly increased in CLP group (0.670 ± 0.017) in comparison with the sham group (0.575 ± 0.027). There were no significant differences in serum creatinine level between phloretin treated (0.646 ± 0.023) and untreated control group (*p* < 0.05), Fig. 1b.

### Serum Creatine Phosphokinase (CPK)

The serum CPK level was not changed significantly when compared to the sham group, Fig. 1c.

### Serum TNF-α levels

**Fig. 2:**
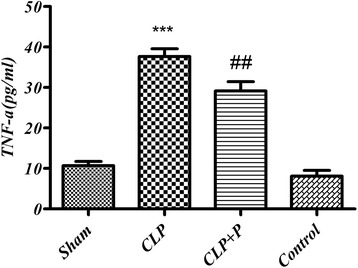
Changes in serum tumor necrosis factor-alpha (TNF-α) in CLP (cecal ligation and puncture) and phloretin treated rats. Results are expressed as mean ± SEM for 7 rats. CLP (cecal ligation and puncture) vs. Sham group (***) *p* < 0.001, CLP+ phloretin vs. CLP group (##) *p* < 0.01

TNF- ***α*** levels were dramatically increased in CLP operated group (37.63 ± 1.92) when compared to the sham group (10.69 ± 1.03), *p <0*.05. This rise was significantly abolished following phloretin treatment (29.14 ± 2.29) *p <*0.05, Fig. 2.

### Liver NF-ĸB level

**Fig. 3:**
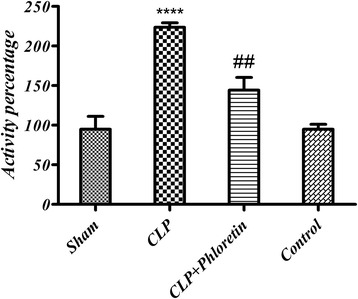
ELISA results of NF-ĸB in liver extract. Bars represent the Sham group NF-ĸB transcription factor as 100 %. CLP (cecal ligation and puncture) vs. sham group (****) *p* < 0.0001, CLP+ phloretin vs. CLP group (##) *p* < 0.01

The protective effect of phloretin on liver NF-ĸB level is shown in Fig. 3. In CLP group, the liver NF-ĸB level was significantly elevated (more than two-fold) in comparison with the sham group (*p* < 0.05), whereas the elevation was significantly reduced in the phloretin treated group.

### Tissue Glutathione (GSH) levels

Sepsis caused a significant decrease (p ≤0.01–p ≤0.0001) in GSH levels in liver, kidney, heart and lung tissues in comparison to the sham group. Phloretin treatment significantly (*p* <0.05) restored the GSH level (Table 1).

**Table 1:** Tissue glutathione level (μmol/g) in septic rats treated with phloretin

Groups	Liver	Kidney	Lung	Heart
Sham	7.08 ± 0.15	5.38 ± 0.09	7.82 ± 0.5	6.38 ± 0.29
CLP	4.87 ± 0.24^***^	3.94 ± 0.27^***^	6.07 ± 0.14^***^	4.94 ± 0.32^****^
CLP + Phloretin	6.08 ± 0.11^###^	4.93 ± 0.17^##^	7.02 ± 0.82^##^	4.81 ± 0.13^##^

Data presented as Mean ± SEM of 7 samples. CLP (Cecal Ligation and puncture) compared to the Sham group. (***) *p* < 0.001, (****) *p* < 0.0001 and CLP+ phloretin vs. CLP group (##) *p* < 0.01, (###) *p* < 0.001

## Histopathological findings

Histological examinations were carried out by a pathologist and pathological events were scored semi-quantitatively. Slides were stained with hematoxylin-eosin and vascular change, local necrosis, hepatocytes morphology, presence and severity of poly morphonuclear leukocytes (PMNL) were considered in the histopathological sections of the liver tissue.

**Fig. 4:**
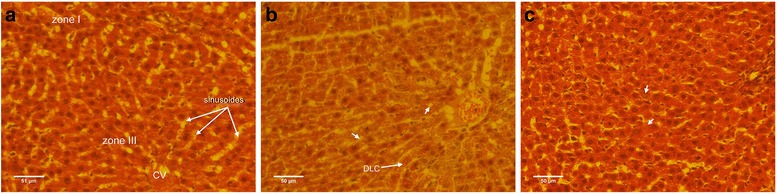
Liver tissue sections are shown. **a**: in the sham group, central vein, normal sinusoids and hepatocytes with regular morphology are seen. **b**: through sepsis group, enormous vascular obstruction, extreme cytoplasmic vacuoles (→), sinusoidal duct dilation, damaged hepatocytes and stimulated Kupffer cells are observed. **c**: in the sepsis group treated with phloretin, mildly damaged hepatocytes (→), moderate vascular obstruction and sinusoidal duct dilatation are noted. Hepatic parenchyma slides were stained with hematoxylin-eosin, original magnification: X400

Normal sinusoids and hepatocytes were noticed in the hepatic parenchyma of the sham group, Fig. 4a. Within sepsis group, serious sinusoidal duct dilation and vascular obstruction, and damaged hepatocytes with many cytoplasmic vacuoles were seen. Moreover, increased number of Kupffer cells and liver dead cells (LDC) were found, Fig. 4b. Meanwhile, treatment with phloretin improved sinusoidal duct dilation, vascular blockage, and hepatocytes morphology. In addition, phloretin decreased the foam like cells near the lobular central vein, Fig. 4c.

## Discussion

Sepsis is described as a severe and irregular systemic inflammatory reaction syndrome (SIRS) against infection, and free oxygen radicals are among important mediators responsible for the inflammatory response. The objective of the present study was to evaluate the protective effect of phloretin against sepsis-induced oxidative organ damage using biochemical parameters, tissue GSH level and histopathological examination of liver tissue.

CLP caused a sharp increase in serum levels of TNF-α in comparison to the sham group (*p* < 0.001). TNF-α is pro-inflammatory cytokine produced by stimulated macrophages [24]. In sepsis, cytokines like TNF-α, interleukin-1 beta (IL-1β) and IL-6 which are released after infection, play an important role in the inflammatory process [25]. Triggering inflammation by high levels of TNF-α may lead to tissue damage and, if not treated, to sepsis or death [26]. Serum levels of TNF-α were significantly suppressed following phloretin treatment as compared to the untreated CLP group (*p* < 0.001). Phloretin is an effective antioxidant for inhibiting the peroxidation of nitroso anions and lipids, and it has antitumor functions [27]. In an animal model, it was demonstrated that phloretin protect hepatocytes against oxidative stress through the ERK_2_/Nrf_2_ pathway and GCL expression [28]. Moreover, it was observed that phloretin has significantly decreased levels of NO, TNF-α, PGE_2_, iNOS and COX-2 in LPS-stimulated RAW264.7 cells [29].

In CLP group, the liver NF-ĸB transcription factor level was significantly elevated (more than two-fold) in comparison to the sham group (*p* < 0.05). NF-κB is a protein complex with DNA transcription regulatory effect. NF-ĸB is a heterodimer and primarily composed of p50 and p65 subunits [30]. The NF-ĸB p65 subunit is phosphorylated and translocated into the nucleus to bind promoter of inflammatory-associated gene and expression inflammatory cytokines and Mediators [31]. It is activated by multiple signals, including tissue damage, oxidative stress, pro-inflammatory cytokines, and pathogen associated molecular patterns, that induce inhibition of kK (IkK) phosphorylation. Stimulated IkK discharge NF-ĸB from its cytoplasmic retainer, an inhibitor of ĸB, facilitating its translocation to the nucleus. NF-ĸB binds to distinct DNA sequences to increase transcription of genes encoding significant inflammatory mediators [32]. Binding of NF-ĸB to DNA, transcription of TNF-α and iNOS are obvious in inflammatory diseases [32–35]. It was observed that phloretin inhibited phosphorylation in MAPK pathways by suppressing NF-κB p65 proteins nuclear translocation in macrophages [29].

Although the exact mechanism of phloretin action remains unclear, a large body of evidence suggests that phloretin exhibits anti-oxidant properties and may be used as an anti-inflammatory agent [29, 36, 37]. Phloretin prevented inflammation process by reducing liver NF-ĸB level (*p* < 0.01). It is believed that phloretin is responsible for NF-ĸB activation mainly via IkK phosphorylation. The IkK phosphorylation inhibits IkK activity which may lead to ĸB attachment to DNA sequences. The following gene expression has been documented in vitro in inflammatory and vascular cells provoked with LPS, staphylococcal enterotoxin A, TNF-α, or IL-1β, and in vivo in models of inflammatory disease [38]. A relation between sepsis and NF-ĸB activation has been reflected in several cell culture and animal studies [38]. Oxidative stress regulates NF-ĸB activation, and the manifest of such stress has been demonstrated in sufferers from sepsis. Previously it was reported that phloretin possess anti-inflammatory properties due to inhibition of nucleus NF-κB translocation and suppression of mitogen-activated protein kinase (MAPK) signaling pathway proteins phosphorylation in human keratinocytes [37, 39]. It is more likely that an increase in NF-ĸB and, therefore, up-regulation of cytokines, will happen in these patients [38]. This is in collaboration with our findings of more than two-fold increase in NF-ĸB transcription factor levels (*P* <0.05) which was significantly decreased by phloretin treatment (*p* < 0.001).

Creatinine level was measured 24 h after sepsis induction and a mild increase in untreated CLP group (0.670 ± 0.017) was observed in comparison with the sham group (0.575 ± 0.027). Creatinine is a byproduct of creatine phosphate in breakdown muscles. Serum creatinine is an important marker of kidney function which is excreted unchanged. In addition, there were no significant differences in serum creatinine phosphokinase level between treated and untreated groups level (*p* < 0.05). Phloretin significantly decreased BUN in comparison to the untreated CLP group. BUN is a good marker for renal damage in cases of decreased Glomerular Filtration Rate (GFR) (suggestive of renal failure).

There is a relation between oxidative stress and peritoneal sepsis [7]. There are several defense mechanisms against the toxic effects of free oxygen radicals. GSH is a natural antioxidant defense system which is found in high concentrations in all cells and in epithelial surface fluid. GSH shows a protective effect by neutralizing free radicals and reactive oxygen intermediates [40, 41]. It is well understood that a wide group of natural products such as plant polyphenols and flavonoids have anti-inflammatory and anti oxidative properties [42, 43]. Polyphenols are the most available dietary antioxidants. A large body of evidence strongly suggests a contribution of polyphenols to the prevention of life threatening disease such as cancers, diabetes mellitus, osteoporosis and cardiovascular [44, 45]. Based on meta-analyses and associated epidemiological studies, polyphenols as secondary plants metabolites are considerably involved in biological defense against ultraviolet radiation or pathogens aggression [46–49]. Flavonoid is a general term for flavanones, flavanols, flavones, chalcones and anthocyanins [50]. Beside antioxidant activity, certain polyphenols possess a potent anticarcinogenic activity through enzyme modulation, upregulation of gap junction communication, gene expression, apoptosis and P-glycoprotein activation in cell culture and animal models [51–53]. Phloretin treatment was able to protect animals against sepsis-induced oxidative tissue damage and maintained GSH levels in hepatic, renal, heart and respiratory tissues in comparison to the untreated sepsis group (*p* < 0.01). It was previously demonstrated that phloretin increases GSH level and HO-1 expression in carbon tetrachloride-induced rat hepatotoxicity [28]. Reduced glutathione as the main source of sulfhydryl pool is well known to be a crucial scavenger of free radicals in the cell [24, 26]. The non-protein sulphydryls binds to a diverse group of electrophilic radicals and metabolites [54]. It has been suggested that antioxidants which save GSH content, may restore the cellular defense mechanisms, inhibit lipid peroxidation and thus provide cellular protection against the oxidative tissue damage. Previously it showed that phloretin has biological and anti-infective activity with beneficial effect on inflammatory bowel diseases (IBD) as well as anti-oxidative charactrisitcs and inhibition of Escherichia coli O157:H7 bio-film formation without harming beneficial commensal E. coli biofilms [15, 55–58]. In addition, phloretin (24 μM) May also be involved in lipid peroxidation and oxidative stress inhibition in cell culture [54].

Erkel et al. showed that dihydrochalcone aglycone phloretin significantly blocked pro-inflammatory gene expression and decreased IL-8, IP-10 and NF-ĸB promoter signal transduction in a dose-dependent manner. Furthermore, the apple juice critically hinder the expression of NF-ĸB regulated pro-inflammatory genes (IL-1β, CXCL9, TNF-α), inflammatory-related enzymes (CYP3A4, COX-2), and transcription factors (STAT1, IRF1) in LPS/IFN-γ provoked MonoMac6 cells without significant influence on the expression of house-keeping genes [37]. Phloretin exhibited anti-inflammatory effects through decreasing IL-6, IL-8, intercellular adhesion molecule (ICAM)-1 production and mRNA expression in TNF-α stimulated HaCaT human cells [39]. Furthermore, neuroprotective effects of phloretin has been demonstrated thorough Nrf2 pathway activation and oxidative stress suppression in rat [36].

## Conclusion

The present study evidenced strong anti-oxidant and anti-inflammatory effects of phloretin against tissue damage from CLP-induced sepsis, likely caused by free oxygen radicals. Thus, it will open a new window for the possibility of clinical application of phloretin in severe conditions such as sepsis. However, further studies required to highlight the exact underlying mechanisms of the observed protective effect. As our knowledge improves in this field, therapeutic targeting would be possible in several disorders.
